# Examining ways to meaningfully support students in STEM

**DOI:** 10.1186/s40594-018-0150-3

**Published:** 2018-12-20

**Authors:** Lisa Martin-Hansen

**Affiliations:** Science Education, College of Natural Sciences and Mathematics, California State University, Long Beach, USA

## Abstract

A strong, positive science, technology, engineering, and mathematics (STEM) identity is a predictor of future career choice in a STEM field. In this commentary, major concepts are explored within and among four different research studies with implications regarding STEM or science identity. This commentary describes ways in which one can view STEM identity as its own construct—and how different experiences affect positive or negative influences upon the formation and continuation of STEM identity. A summary of external and internal factors is included with discussion of the pertinent points regarding facilitation and development of STEM identity within educational settings.

When I started out being excited by science — but seeing that there weren’t any women scientists — I thought I had no prospects whatsoever. (Joan Argetsinger Steitz, recipient of the 2018 Lasker-Koshland Award for Special Achievement in Medical Science (Thomas 2018))

Recruitment and retention of individuals into science, technology, engineering, and mathematics (STEM) majors in college evokes a student’s STEM identity—how a person views herself in terms of being a mathematician, scientist, engineer, or perhaps a computer programmer (Seyranian et al. 2018; Vincent-Ruz and Schunn 2018). If one cannot image herself in that role, then it is less likely choices will be made leading to a STEM career—no matter how motivating or engaging a STEM activity might be. Education should play a role in fostering positive STEM identity as experiences in K-12 education can impact perceptions students have about themselves (Vincent-Ruz and Schunn 2018; Vongkulluksn et al. 2018). However, there are confounding issues in learning and the learning environment such as student-student interactions, instructor-student interactions, and other social interactions outside of the school setting such as parents, friends, and other persons that can impact identity. Inevitably, some of these interactions may be influenced by biases. It is necessary for instructors to learn about these factors so more positive learning environments can be created for STEM learners (Vincent-Ruz and Schunn 2018).

While it is easy to say that we need to increase the diversity in STEM fields, specifically to include more members of underrepresented groups in STEM including women and people of color (Archer et al. 2014; McGee and Bentley 2017; Brotman and Moore 2008; Clark Blickenstaff 2006), the reality of the situation is that enacting that goal is more complex.

Learning in STEM domains has often consisted of memorization of facts devoid of deep connection, separated from life and often failing to integrate the STEM disciplines (Momsen et al. 2010). This learning seems to take place in an environment with little attention to different modes of communicating and meaning making—after all, it is as if people are saying, “Aren’t these universal ideas? Shouldn’t presenting these ideas simply be enough?” Individuals are left to make meaning and to find ways to create personal connections to deeply internalize this knowledge. What scholars are finding is that although concepts within fields may initially be seen as universal and culturally benign, the fields of science, mathematics, technology, and engineering are human endeavors (Franklin 1995) sifted through our lenses of personal experiences. The investigations, sense-making, creativity, and representation of ideas and communication of these ideas are all conducted by humans and therefore have human implications.

Further, individuals’ cultures and belief systems are at play at all stages of inquiry and problem-solving (Aikenhead 1996). Therefore, the way we interpret what we hear or see in a classroom includes scientific or mathematical ideas, as well as hidden aspects such as the following: who is doing the teaching? How am I able to engage in learning? Are my ideas valued as I wrestle with the material? Am I allowed to speak as often as someone else of a different gender or culture?

The question of “am I seen as someone who could be a scientist, engineer, computer scientist or mathematician” has been tackled by various scholars (Kim et al. 2018; Aschbacher et al. 2010) because individuals’ sense of whether or not they belong in a particular field can also impact their choice of a discipline or career. Issues have arisen when students do not see themselves represented in a particular STEM field. Without appropriate role models, they have difficulty imagining someone who looks or speaks like them in that field (Cole and Espinoza 2008).

## The interplay of internal and external experiences upon STEM identity

In this commentary, I focus on three themes relevant to science education. The overarching theme of the special issue involves science identity, and each study either measures or enacts activities that impact science identity in various ways. My discussion is organized into three themes that emerged from the contributions to this special issue: (1) encouraging recruitment and retention through science identity, (2) participation in science projects and courses to increase proficiency in science, and (3) the impact of mentorship on students.

## Identity relating to recruitment and retention

The research literature in STEM education has focused quite a bit on science interest with less emphasis given to science identity (Vincent-Ruz and Schunn 2018). However, the limited previous research on identity has found that a strong science identity predicts who will pursue a science career (Chemers, Zurbriggen, Syed, Goza, & Bearman, 2011; Robnett and Leaper, 2012). Developing a deeper understanding of science identity is needed to determine the degree to which a particular science identity might be connected to other specific, desired outcomes. After conducting their studies, it is clear that several of the authors in this special issue find that a positive STEM social identity is necessary if students are to be recruited and retained in STEM fields (Robnett et al. 2018; Seyranian et al. 2018; Vincent-Ruz and Schunn 2018; Vongkulluksn et al. 2018). The investigations into science identity show that it is possible that science identity is as important as science interest in whether or not a student will engage in STEM learning.

The authors investigating science identity focused upon the idea that identity “is built from internalization from our experiences and socially constructed with others” (Vincent-Ruz and Schunn (2018). They stated that research has shown that there are three areas that drive science identity including (1) a *match between school science and real science*, (2) *consistent extrinsic and intrinsic motivation*, and (3) *a sense of community and affiliation* (Vincent-Ruz and Schunn 2018, p. 2).

Interestingly, in their investigation, Vincent-Ruz and Schunn (2018, p. 2) found that not only are there key components of science identity for middle and high school students that are distinct from other attitudinal constructs, but there are also ways in which science identity can predict students’ choices. Science identity, when positive, was related to the number of science activities the student chose to do. Sadly, but not surprisingly, middle school and high school females displayed lower science identity as measured on the science identity assessment. That correlated positively with less participation in science activities.

Another finding was the importance of external and internal factors of science identity. For instance, context (an external factor) is important and affects students’ internalization of science identity (Vincent-Ruz and Schunn 2018). Perceptions of external recognition of science identity and internal perceptions of science identity *both* play a role in choices students make in optional science activities. This means that influential others could be a catalyst in changing students’ self-perceptions. This is a promising finding in that it points to the value and possible impact of thoughtfully created activities resulting in a positive experience that was led by someone who is seen as influential—a scientist, teacher, influential peer, or a mentor.

## Participation in science

As researchers have determined that context plays a large role in the development of science identity, we should consider the environment in which students are learning. Specifically, what types of activities and interventions and how do they affect science identity—whether it be a positive or negative effect (Vincent-Ruz and Schunn 2018). The environment, or context, in which a student is placed could be altered to include activities designed to foster experiences of success, thereby leading to greater science identity.

Research literature has shown that women are underrepresented in physical science and several factors contribute to this situation. Some of these factors are published in the compilation of research entitled *Why so few? Women in science, technology, engineering, and mathematics* (Hill et al. 2010). These include helping our students to be aware of the potential impact of stereotype threat and implicit bias as well as the need for a growth mindset instead of a fixed mindset of abilities or talents. Efforts to provide interesting experiences for girls in STEM, supportive environments for undergraduate women in science and engineering, and the opportunity to reflect on and assess possible implicit bias positively affected women in STEM.

Researchers found that women did not feel the same sense of belonging in physics as they did at the university as a whole (Seyranian et al. 2018). They also felt more *uncertainty* in physics classrooms. In contrast, men felt they belonged at the university in general and in physics more or less *equally.* It is possible that the reason why there was less of a feeling of belonging in the physics classroom versus the university was due to an unequal representation of women and men in the physical sciences (Hill et al. 2010) as this is a typical situation in many university courses. In fact, 77% of the physics students in the Seyranian et al.’s study were identified as male.

Gender differences were present in physics identification and physics academic achievement as measured on the Force Concept Inventory*.* Other issues at play include possibilities of stereotype threat on standardized assessments (Hill et al. 2010). Women who were less likely to identify with physics were also less likely to do well on the Force Concept Inventory assessment (Seyranian et al. 2018). In contrast, in the same course, women and men earned similar grades. Further, men identified more with physics at the beginning and end of the course than women. A bright point is that although stereotype threat can negatively impact how women proceed in a physical science trajectory, data also shows that higher physics identification for women can result in MORE flourishing over time (Seyranian et al. 2018).

Another way to consider the context other than how genders are represented in the classroom is to examine the impact of learning tasks upon students’ STEM identity. Researchers have found that by increasing proficiency in STEM, positive STEM identity can be increased as well (Vongkulluksn et al. 2018). Given this possible link, providing experiences where students are more autonomous and experience success in STEM, instructors can positively impact students’ feeling of belonging and STEM identity.

Design-based makerspaces have the possibility for developing proficiency in STEM by providing authentic science inquiry engaging students through exploratory investigations, collaboration, and technology use (Rivera Maulucci, Brown, Grey & Sullivan, 2014; Krajcik, Blumenfeld, Marx, Bass, & Fredricks, 1998). Vongkulluksn et al. (2018) found that makerspaces can provide greater autonomy and authentic engagement in inquiry in order to solve a problem. However, these spaces need to be carefully monitored with appropriate scaffolding to encourage successful task completion. In one study, while students participated in actions very relevant to their lives, most chose projects that were more complicated than originally thought. When they realized that they needed to modify their plans and lower their expectations, there were negative emotions associated with the project. The negative emotions also correlated with the decline in self-efficacy in STEM. Despite this decline, levels of situational interest and self-efficacy remained fairly high. It is possible that, with different guidance, the students may have been able to choose a project that was more achievable or they could have chosen to complete a small component of the larger design, thereby experiencing success along the way. A lesson learned was that if activities are not structured appropriately, negative experiences in the design-based makerspace can then negatively impact STEM self-efficacy and situational interest.

It is important for instructors to be aware of both the impact of unbalanced representation of students (in gender and ethnicity) as well as how structure and scaffolding of the learning process can impact STEM identity. When instructors offer scaffolds for efficacy and experiences of success, there is an opportunity to develop students’ self-efficacy, therefore affecting STEM identity. Ideally, these interventions could be used to develop complex problem-solving skills, as such skills are needed to thrive in a world where one must integrate cognitive, social, and communication skills (National Research Council 2010).

## Importance of mentorship in science

A third theme emerging from the four articles is that of the impact and importance of mentorship. There is evidence that the role of a mentor or an influential person can have an impact on students’ STEM identity (Robnett et al. 2018). Having a mentor is particularly important for students who are at risk for stereotype threat. Stereotype threat happens when a common stereotype affects performance. For instance, since it is commonly thought that women struggle in math, thus an additional cognitive and emotional burden weighs down upon many women when taking an assessment in mathematics (Spencer et al. 1999). Women are also typically harder on themselves when assessing their abilities in traditionally male fields like math or physical sciences. Women hold themselves to higher standards in terms of their interpretation of success in a course. For instance, when respondents were asked, “How high would you have to score to be convinced that you have high ability at this task?”, women required a higher score of themselves than did men (Correll 2004, p. 106).

Strategies used to offset this effect include the inclusion of female role models in the particular STEM fields for positive prototypes. College environments are very important, as that is where students may see women role models for the first time in their academic careers. In a study of mentors and mentees, Robnett et al. (2018) examined *instrumental* (skill-based, task-focused guidance) and *socio-emotional mentoring* (general support and guidance) and how those mentorship types impacted undergraduate college students. In this study, information was obtained from both undergraduates and their mentors.

Positive effects were found of mentor-mentee relationships on all genders and ethnicities for both instrumental and socio-emotional types of mentorship (Robnett et al. 2018). When negative mentorship (such as ignoring the mentee) was reported, there was also negative impact on science identity. Instrumental mentorship had the largest positive impact upon science identity. This suggests that skill-based, task-focused guidance from mentors is especially important. Of interest, as data was obtained from both the mentor and the mentee, the authors indicated that there were nuances and insights provided by the mentors that would likely have been lost had only the mentees been surveyed.

While it is unclear if it was the mentorship that had the direct effect of increased (or decreased) science identity, there was an indication that the relationship between the mentor and mentee is an important one. Those students who had the highest science identity also reported receiving more instrumental and socioemotional mentoring. Overall, the study underscores the importance of instructor awareness of possible negative stereotypes about women (and other underrepresented groups in STEM) to help counteract an impulse to respond in a negative fashion (Devine et al. 2012).

## Discussion and recommendations

Based on the articles reviewed in this special issue, the development of a positive science identity requires time and effort from the individual as well as from individuals in their educational environment. Positive science identity can then in turn have a positive impact on science graduates who would ultimately join the workforce. There is a strong indication that the context of science learning has a large impact upon students’ identities and later participation in science.

## Internal and external factors impacting STEM identity

Individuals’ STEM identities have been found to be impacted by both internal and external factors. As STEM identity is linked to later success in STEM courses and programs, identity is important to examine (Vincent-Ruz and Schunn (2018). Conversely, if students’ experience success in STEM activities, there is a greater chance of developing positive STEM identity and agency (Vincent-Ruz and Schunn 2018; Vongkulluksn et al. 2018).

Internal factors include the way that females view themselves within a particular setting—do I fit in or not? Even with similar grades, females can still feel that they are outliers. This was noted in educational settings, such as physics classrooms, when the room is dominated by male peers and male instructors. These male-dominated settings may promote a perceived stereotype threat of men’s possible superiority in physics and mathematics achievement. The stereotype threat is often also at play when students take standardized tests. Male dominated STEM classrooms could results in poorer performance for women on standardized assessments due to experiencing stereotype threat in the testing situation. This points to the importance of having young women be aware of stereotype threats and providing inoculations against threat such as telling examinees that men and women actually have equal ability and are expected to perform comparably (Hill et al. 2010; Vincent-Ruz and Schunn 2018).

If students struggle with tasks and concepts within a STEM setting, those experiences can negatively affect their self-efficacy; therefore, tasks need to be carefully chosen with appropriate scaffolding provided so that students are not overwhelmed with seemingly impossible goals. It is important for the instructor to be a guide and facilitator to help students determine whether inquiry investigations or projects are appropriate and students define goals that can be accomplished (Vongkulluksn et al. 2018).

Externally, several factors could affect STEM identity. The instructor is key in that she or he creates learning experiences that can positively affect STEM identity—such as inquiry projects tied to authentic problems and allowances for autonomy in designing an investigation. These types of experiences were found to positively impact a students’ STEM identity for both males and females (Vongkulluksn et al. 2018).

Conversely, if a class is filled predominately with males, females report that they feel less likely that they belong in that particular course or field of study. In those cases, a mentorship program can help to alleviate some of these issues—especially if mentors provided both instrumental (practical “how to” sessions) as well as socioemotional mentoring as both of these were tied to higher scientist identity (Robnett et al. 2018).

Looking to other research literature in institutional transformation, the Meyerhoff Program proposed a social transformation theory of change (Maton 2008) that combines an empowering settings theory with knowledge about change on college campuses to support inclusive excellence (Williams, Berger, & McClendon, 2005). The Meyerhoff program has had promising results in recruiting, achievement, and retention of minority students in STEM. The suggestions made by those involved in the Meyerhoff program align well with the findings from the studies in this special issue examining STEM identity. The Meyerhoff program researchers (Maton et al. 2012) summarized the mechanisms for success including the development of empowering settings for minority student achievement, larger institutional change processes, and assessment and evaluation.

Their participants were five times more likely to graduate with a Ph.D. in a STEM field compared to other African American students not participating in the program. According to Maton et al. (2012), the Meyerhoff Scholars Program’s impact was due to the eventual redesign of introductory science courses for all students. This led to the implementation of an “institution of inclusive excellence” for all (Maton et al. 2012, pg. 12). The aspect of *inclusiveness* in the introductory science courses relate to the findings regarding social identity and belonging that are key components in STEM student success and retention. They focused on programmatic means to enhance minority student achievement through the development of empowering settings along with the larger institutional change process. In this way, they worked on the external components that were part of the context of their learners. They found it was necessary to provide support to enact the strategies listed above to bring about necessary change in the larger institutional environment.

## Professional development

Understanding science identity and implementing actions to positively impact science identity is crucial for learning and recruitment of diverse students into science, technology, and computer science, mathematics, and engineering careers. Science educators are best positioned to impact individuals as they can become aware of these research findings that can influence new and existing initiatives addressing science identity, social belonging, and achievement in science.

Professional development for educators is important as it is likely that our instructors are not fully aware of what they can do to positively impact students’ science identities. For instance, knowing that self-doubting women who are also high achievers typically possess a low identity as a scientist (Robnett & Thoman, 2017) can help a mentor to consider what actions to take with her or his mentee. As we consider types of mentorship in science fields of study, it is possible that the student outcomes in science majors and careers may be influenced by “the mentor’s approach to managing conflict such as constructive and culturally sensitive versus critical and culturally insensitive” (Robnett et al. 2018, p. 12). As mentored research apprenticeships are often included in academic outreach programs that aim to promote diversity in science fields, this is an area to examine more fully.

Seyranian et al. (2018) argued that “Classroom climate may be a critical component of helping retain women in STEM, particularly for universities that have a predominately commuter student population such as the current sample, where only 9% live on campus” (p. 9). Part of classroom climate has to do with how well students feel that they fit into the field of study and in the classroom itself. Researchers found that a sense of belonging was a necessary component related to flourishing and was shown to be an issue for women and for both genders from underrepresented ethnic and racial groups in science. An instructor’s teaching behaviors are also crucial. For instance, are women asked to participate in discussions and projects? If not, strategies can be put into place to more effectively involve all learners within the classroom—whether that is increased wait-time before calling on students to answer questions, or purposefully organized student working groups.

In general, it is critical that educators and mentors have training regarding STEM identity and the impact of STEM stereotypes on mentors and mentees (Kim et al. 2018). Mentees and students are affected by the signals and words that instructors share with them. It would be helpful for mentors to realize that females have high expectations of themselves, more so than males, when it comes to their perception of what it means to be proficient in an area of study (Hill et al. 2010); therefore, encouragement of young women is necessary when they feel that they are struggling.

## Hiring practices

Another strategy to increase inclusiveness, identity, and belonging in science is the importance of hiring a diverse teaching pool. Simple underrepresentation can negatively affect a social identity in physics where the student asks him or herself, “Do I belong here?”

There are indications in the Seyranian et al. (2018) as well as in other research literature (Stout et al. 2011) that female mathematics and science professors have a positive impact upon undergraduate women who take their courses. Women in those courses also are more likely to describe themselves as being successful in the future in a STEM career and to associate women with leadership (Stout et al. 2011). As indicated earlier, if a student has more of a positive identity or social identity in a particular field, that then relates to more success in coursework.

It should be a human right to have equal access to education and education that supports *all*. We also need to be certain that those of us who are teaching science and engineering have high expectations for all (Kim et al. 2018). That requires checking our implicit biases and how those implicit biases can impact our perception of our students as well as their subsequent perceptions of themselves because of our actions. These studies indicate that STEM social identity has important implications for attempts to grow the diversity of the STEM professionals. By paying close attention to the actions, initiatives, and structures of education and how these intersect with STEM identity, we can begin to make a substantive impact creating access, opportunity, and support for contributions of all peoples in STEM fields.
